# Comparison between use of a pleural drainage system with flutter valve and a conventional water-seal drainage system after lung resection: a randomized prospective study

**DOI:** 10.1590/1516-3180.2023.0224.R1.08022024

**Published:** 2024-04-22

**Authors:** Rodrigo Caetano de Souza, Lilianne Louise Silva de Morais, Mario Claudio Ghefter, Juliana Pereira Franceschini, Fernando Campos Gomes Pinto

**Affiliations:** I MD, MSc. Thoracic surgeon, Preceptor of the Residency Program in Thoracic Surgery, Hospital do Servidor Público Estadual de São Paulo (IAMSPE) Francisco Morato Oliveira, São Paulo (SP), Brazil.; II MD, Thoracic surgeon, Hospital do Servidor Público Estadual de São Paulo (IAMSPE) Francisco Morato Oliveira, São Paulo (SP), Brazil.; III MD. Thoracic surgeon, Director of the Thoracic Surgery Service, Hospital do Servidor Público Estadual de São Paulo (IAMSPE) Francisco Morato Oliveira, São Paulo (SP), Brazil.; IV PhD. Physiotherapist, Project Leader, ProAR Foundation, São Paulo (SP), Brazil.; V MD, PhD. Neurosurgeon, Head of the Cerebral Hydrodynamics Group, Functional Neurosurgery Division, Institute of Psychiatry, Hospital das Clínicas, Faculty of Medicine, Universidade de São Paulo (USP), São Paulo (SP), Brazil.

**Keywords:** Pleural Cavity, Thoracic Surgical Procedures, Lung, Pleural effusion, Postoperative period, Time in hospital

## Abstract

**BACKGROUND:**

There is still a debate regarding the most appropriate pleural collector model to ensure a short hospital stay and minimum complications.

**OBJECTIVES:**

To study aimed to compare the time of air leak, time to drain removal, and length of hospital stay between a standard water-seal drainage system and a pleural collector system with a unidirectional flutter valve and rigid chamber.

**DESIGN AND SETTING:**

A randomized prospective clinical trial was conducted at a high-complexity hospital in São Paulo, Brazil.

**METHODS:**

Sixty-three patients who underwent open or video-assisted thoracoscopic lung wedge resection or lobectomy were randomized into two groups, according to the drainage system used: the control group (WS), which used a conventional water-seal pleural collector, and the study group (V), which used a flutter valve device (Sinapi^®^ Model XL1000^®^). Variables related to the drainage system, time of air leak, time to drain removal, and time spent in hospital were compared between the groups.

**RESULTS:**

Most patients (63%) had lung cancer. No differences were observed between the groups in the time of air leak or time spent hospitalized. The time to drain removal was slightly shorter in the V group; however, the difference was not statistically significant. Seven patients presented with surgery-related complications: five and two in the WS and V groups, respectively.

**CONCLUSIONS:**

Air leak, time to drain removal, and time spent in the hospital were similar between the groups. The system used in the V group resulted in no adverse events and was safe.

**REGISTRATION:**

RBR-85qq6jc (https://ensaiosclinicos.gov.br/rg/RBR-85qq6jc).

## INTRODUCTION

Chest drains are essential in the post-operative period of lung resection because they enable air escape and the drainage of secretions produced by surgery.^1^

Conventional pleural drains with a single bottle using a water seal became the standard for this purpose at the beginning of the 20^th^ century and are currently the most used devices worldwide.^2^ Keeping them in place for longer periods frequently limits the time of hospital discharge and directly influences the cost of hospital stay as well as the anxiety levels of patients and their caregivers.

There is still a debate on the most appropriate pleural collector model to ensure a short hospital stay and minimum complications, principally considering the reality of health services in developing countries.

The need to reduce adverse events related to pleural drains, and to make them easier to use, motivated Heimlich, in 1968, to create a one-way valve for this purpose, which is referred to by his name.^3^ It is a mechanical device, designed to isolate the external environment from the sub-atmospheric intrapleural environment.^4,5^ Since then, other drainage systems have been developed that aim to replace water-seal drains and make hospital stays shorter.^6^

In view of the options currently available, there is a need to identify systems that are easy to handle, reduce the time for which the pleural drain needs to remain in place, can be emptied, have a rigid reservoir, are low cost, are perceived by the patient as safe, and enable suction when necessary.^4,7^

## OBJECTIVE

This study aimed to compare the time of air leak, time to drain removal, and length of hospital stay between two systems: (a) a standard water-seal drainage system and (b) a pleural collector system employing a unidirectional flutter valve and a rigid chamber after lung resection and to report any complications related to patients and/or the systems.

## METHODS

This randomized clinical trial included patients who underwent open or video-assisted thoracoscopic lung resection surgery (VATS) at Hospital do Servidor Público Estadual Francisco Morato de Oliveira (IAMSPE), São Paulo, Brazil, between October 2020 and May 2022. The same surgical team performed all the surgeries.

The study was approved by the Ethics and Research Committee of IAMSPE on February 11, 2020, under protocol no. 3.832.730. All patients signed an informed consent form prior to enrollment.

Patients included in the study were adults aged >18 years indicated for elective lung wedge resection or lobectomy, with predicted post-operative forced expiratory volume in one second (FEV_1_ppo) ≥ 40%. Patients with any type of present or past pleural infection, those needing concomitant chest wall resection, those presenting with diffuse adhesions in the intraoperative period, and those who died before the chest drain was removed were excluded from the study.

Air leaks were controlled by mechanical stapling of the fissure (Echelon Flex, Ethicon, Johnson & Johnson^®^) and manual sutures. In all surgeries, only one tubular pleural drain, nº 28 Fr, was used.

Patients were randomized into groups, according to the drainage system to be used: 1) the control group (WS), which used a conventional water-seal pleural collector, and 2) the study group (V), which used a flutter valve device, the Sinapi^®^ Model XL1000^®^ pleural collector employing a flutter valve.

The decision on which drainage system to use was randomized using the randomization calculator available on the website *calculatorsoup.com*, allocating participants randomly to the V or WS groups. The surgeon was informed of the type of system to be used only after the completion of the surgical procedure.

**Figure 1.**
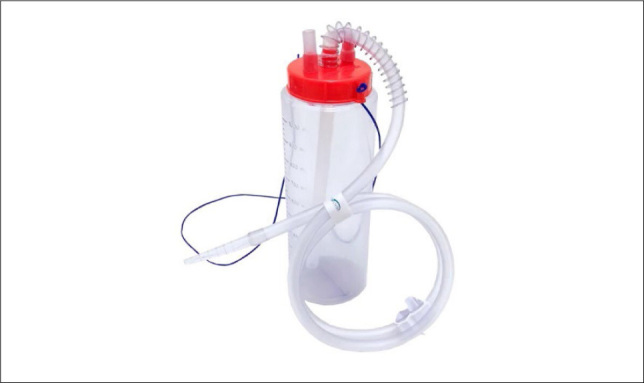
Water seal pleural collector comprised a polyvinyl chloride (PVC) bottle with volume of 2L, under an initial sterile water seal of 2 cm.

In the WS group, the pleural collector comprised of a polyvinyl chloride bottle with a volume of 2 L, under an initial sterile water seal of 2 cm (**Figure 1**).

**Figure 2.**
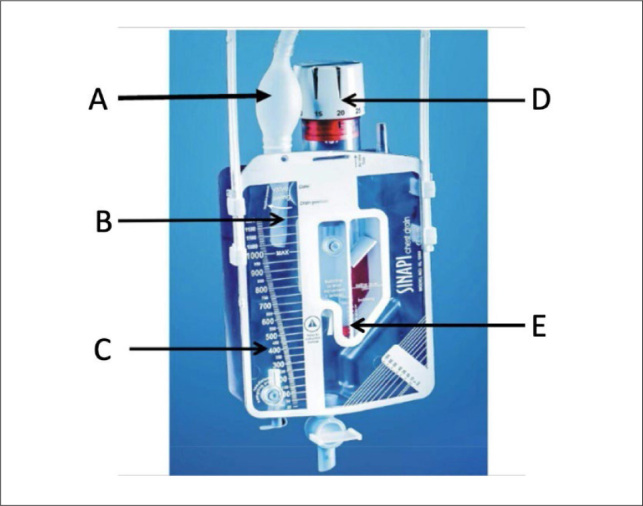
Pleural collector with valve. A: suction bulb; B: one-way flutter valve; C: calibrated liquid collection chamber; D: suction regulation valve; E: level scale for checking air bubbles.

In the V group the pleural collector was a chamber in the form of a rectangular transparent rigid prism of acrylonitrile butadiene styrene polymer, with numbering on its external surface to identify the volume stored and an internal capacity of 1,000 mL. This had a half-turn type evacuation nozzle on the interior surface. It was connected to a tubular drain using a silicone tube with an adapter for various drains of different calibers. Connected to the silicone tube, in the initial part of the rigid prism, was a one-way mechanical anti-reflux ‘flutter’ valve, with the same format and effect as the Heimlich valve. The flap in the flutter valve was made of an anti-adherent polymer. Additionally, a compressible suction valve made of silicone was connected to the end of the connection tube of the drain close to the flutter valve. When pressed, this bulb created a vacuum in the drain system, allowing air leaks to be detected. If the bulb was squeezed and did not immediately return to its resting inflated position, it was assumed that there was no air leakage through the pleural drain. (**Figure 2**).

All patients underwent anteroposterior chest radiography within 24 hours post operation to check for pneumothorax or pleural collection. Patients whose radiographs showed a residual pleural space in the post-operative period underwent suction using the drainage system.

In the WS group, pleural suction of 10 cmH_2_O was applied (maximum capacity in that common bottle) with the standard system of three bottles. Patients in the V group who presented with residual pleural space or for whom the bulb did not remain compressed after compression were assumed to have residual air leaks and underwent pleural suction of 20 cmH_2_O.

Air leaks were considered to be stopped:

- In the WS group, when there was no air bubbling through the water seal during coughing with exertion.
- In the V group, when the bulb remained collapsed for 10 minutes after compression, indicating air leaks were completely absent.

In both groups, the drain was removed when (i) there was no air leak, (ii) the liquid drawn was <200 mL in 24 hours, and (iii) a prior chest radiograph showed complete lung expansion.

A further chest radiograph was obtained at the return outpatient visit seven days after hospital discharge.

The following variables were recorded:

- Time of air leak (TAL) (days);
- Time to removal of drain (TRD) (days);
- Time in hospital (TIH) (days);
- Total liquid drained during the entire hospitalization period (TLD) (mL);
- The number and type of post-operative complications; and
- Any dysfunctions related to the drainage system.

The following were considered complications related to the patients: empyema, subcutaneous emphysema, hospital discharge with a Heimlich valve, readmission to the hospital, re-draining, or prolonged air leak (defined as an air leak through the drain for six days or more). The following complications were considered system-related: prolonged clamping, empty water-seal system, mechanical valve clogging, and unintentional disconnections.

The sample size was determined based on the descriptive statistics of a pilot study, adopting an alpha of 0.05 and a beta of 0.20, which indicated the need to evaluate a minimum of 61 patients.

The data are presented using descriptive statistics, average and standard deviation for quantitative variables, and absolute numbers and relative percentages for categorical variables.

The quantitative variables were compared between the groups using the Independent Samples T Test for the variables age and VEF_1_ppo and the Mann-Whitney test for the variables total drain volume, TRD, TAL, and TIH. Categorical variables were compared using Pearson’s chi-squared test. All analyses were performed using R software (version 4.2 for Mac iOS), with a significance level of 5% (P < 0.05).

Sinapi^®^ model XL1000^®^ pleural collectors were donated by the manufacturer (Sinapi Biomedical, Idasvallei, Stellenbosch, 7599, South Africa). Although the manufacturers supplied the device, they had no interference of any form during the study.

## RESULTS

The study sample comprised 64 patients. One patient who died before drain removal was excluded. Therefore, the data from 63 patients were analyzed: 31 and 32 in the WS and V groups, respectively. **Table 1** shows the demographic, clinical, and surgical characteristics of the study participants. There was a small predominance of women – 33 patients (52%), with age varying from 18 to 81 years (average age: 65 ± 13 years). There were no significant demographic differences between the two groups.

**Table 1.** Demographic, clinical, and surgery characteristics of the total sample and the two groups

Variables	Total n = 63	Control group (Water seal) n = 31	Study group (Valve) n = 32	P
Gender				0.80*
Female	33 (52)	17 (53)	16 (50)	
Male	30 (48)	14 (47)	16 (50)	
Age (years)	65 ± 13	67 ± 9	63 ± 16	0.70☨
VEF_1_ppo (mL)	67 ± 15	66 ± 13	68 ± 17	0.70☨
Diagnoses				0.48*
Lung cancer	37 (63)	21 (70)	16 (55)	
Granuloma	6 (10)	2 (6.7)	4 (14)	
Metastasis	5 (8.5)	3 (10)	2 (6.9)	
Blebs	2 (3.4)	0 (0)	2 (6.9)	
Hamartoma	3 (5.1)	2 (6.7)	1 (3.4)	
Other	6 (10)	2 (6.7)	4 (14)	
Type of surgery				0.31*
Wedge	33 (52)	14 (45)	19 (59)	
Lobectomy	30 (48)	17 (55)	13 (41)	
Type of surgery				0.45*
Open surgery	29 (46)	15 (48)	13 (41)	
VATS	34 (54)	16 (52)	19 (59)	
Total liquid drained (mL)	400 [200-1100]	425 [115-1100]	350 [200-1350]	0.46☦
TAL	2 [1-4]	2 [1-3]	2 [1-4]	0.81☦
TRD	3 [2-5]	4 [3-6]	3 [2-4]	0.08☦
TIH	4 [2-6]	4.5 [3-7]	3 [2-5]	0.11☦
Complications	7 (12)	5 (16)	2 (6.2)	0.26*
Deaths	4 (6.2)	2 (6.2)	2 (6.2)	0.90*

* Pearson Chi-squared test (²)

☨ T-test for independent variables

☦ Mann-Whitney test.

SD = standard deviation; VEF1ppo = predicted post-operative forced expiratory volume in one second; VATS = video thoracoscopy; TAL = time of air leak (days); TRD = time to removal of drain; TIH = time in hospital (days). ^a^Amounts expressed as n (%), average ± standard deviation, or median [interquartile interval].

The majority (63%) of patients had lung cancer, and there was no difference in the diagnoses between the two groups. The most commonly performed surgery was wedge resection (52% of the cases, 45% in the WS group and 59% in the V group). Thirty-four surgeries were performed using VATS with a similar distribution between the two groups. Pleural suction was applied in 18 patients: four (6%) in the WS group and 14 (21%) in the V group.

The TAL and TIH were similar between the two groups. TAL varied from one to 20 days, with a median of two days. TIH varied from one to 23 days.

The TRD was similar between the two groups, with the median time for removal being four days [Interquartile range(IQR) 3–6] in the WS group and three days [IQR 2–4] in the V group; however, this difference was not statistically significant.

Four patients died after drain removal (two in each group). The causes of death were pneumonia due to Coronavirus disease ( COVID-19) in three cases and massive lung thromboembolism in one case.

Seven patients presented with surgery-related complications: five in the WS group and two in the V group (Table 1). There were a total of 14 complications in these patients; in the V group, each patient had one complication, and in the WS group, the patients presented with two or three associated complications. The most frequent complications were prolonged air leak (in four patients) and empyema (in three patients). All cases of prolonged air leaks were observed in the WS group. Furthermore, hospital discharge with the Heimlich device in place, readmission, re-drainage, and drain malfunction were observed in the WS group.

## DISCUSSION

In this study, no significant differences were observed in TAL, TRD and TIH between the two collectors evaluated, although the medians for TRD and TIH were lower in the V group. Flutter valves have been used as an option for reducing TIH for patients who have undergone surgery;^5^ however, other studies with a one-way valve have not shown any difference in TRD when compared with a water seal.^5,7-10^

Pleural suction was used more frequently in the V group than in the WS group. This is explained by the fact that suction was applied to the WS group only when the chest radiograph showed incomplete expansion. Chest radiography may fail to diagnose pneumothorax in up to 39% of cases when compared to chest ultrasound examination.^11^

Suction was applied in a larger number of patients in the V group, since the bulb of the drainage system, with its function of compression followed by observation of whether it inflated again, helped in the evaluation of complete expansion. The valve system used in this study enabled a more objective assessment of air leakage using a silicone vacuum bulb.

In the WS group, pleural suction was performed using three bottles. In this system, there was a limit of 10 cm for the difference in the levels of liquid, indicating suction pressure. In the V group, suction at 20 cmH_2_O was easily established using a numbered pressure regulator.

Dysfunctions of water-seal pleural chest drains are known to occur and have been reported in the literature. Previous studies have described inadequate connections, low volume of liquid in the bottle, loss of connection between the drain and the chest tube, kinks in the system’s connecting tube, inadequate clamping, blockage of the air output relief valve, failure in suction, and consequences of placement of the system above the level of the chest.^12,13^ In our study, there was one dysfunction in the water-seal system: the patient, in a moment of confusion, emptied the collector, which led to lung collapse, prolonged air leak, and empyema.

One experimental study^6^ showed that a flutter valve is physiologically more efficacious than a water seal for treating post-operative air leaks. This is partly explained by the fact that with the valve, there is no movement of the lungs against the chest wall, which occurs with the water seal through the movement of the liquid in the bottle. Other authors^14^ considered it possible that the use of the flutter valve, in isolation or using malleable collector bags, was discouraged in some cases owing to the presence of adverse events as well as the absence of a rigid reservoir, and thus the possibility of suction. One study reported complications such as hypertensive pneumothorax due to blockage of the valve.^15^ Another study comparing the use of a valve and a water-seal collector for lung surgeries reported two cases of valve obstruction by blood clots, one disconnection of the valve, and the need to change the water seal in one patient.^16^

The materials used to manufacture valves have evolved. In a randomized study published in 2006,^7^ which compared the TRD between a water-seal system and a new flutter valve model in patients who underwent pleural drainage following blunt and perforating lung trauma in a referral hospital in South Africa, no clogging by blood was reported with the flutter-valve system. In our study, no adverse events were directly associated with the flutter-valve drain system.

In addition to the systems used in this study, there are now digital systems that enable a numerical and objective evaluation of the air leak flow and the amount of liquid withdrawn.^17^ These tend to be lighter than the common water-seal system; however, these are not emptiable, and their standard reception canister has a maximum volume of 800 mL. In addition, these require a non-disposable electronic pump for functioning, which costs approximately US$ 4,000.

Patient access to various types of pleural drainage systems is not uniform because ex-factory and retail prices vary between the types of devices and where they are produced, duty, and taxes. As illustrative examples: (i) the ex-factory price of the water-seal pleural drain with a simple bottle (used in the WS group) is approximately US$ 7; (ii) the ex-factory price of the Sinapi^®^ XL 1000 valve system (used in the V group) is approximately US$ 20; and (iii) the ex-factory price of the disposable canister and the connecting tubes of the digital drain is approximately US$ 40, and this system requires a permanent pump. With taxes, charges, and other costs, these values could increase significantly.

Notably, the data used in this study were collected during the COVID-19 pandemic. Elective surgeries were interrupted for four (non-consecutive) months during this period, and COVID-19 was a factor in the mortality that occurred in this study. Of the four deaths observed, three were directly related to COVID-19 and tested positive in the post-operative period after the appearance of symptoms. The fourth patient died because of acute thromboembolism. A relationship with COVID-19 was suspected, although not confirmed, possibly because of limitations in the interpretation of diagnostic methods at the beginning of the pandemic. Other studies have reported an increase in mortality due to COVID-19 following chest surgery.^18,19^

## CONCLUSION

In conclusion, the results for air leak, time to drain removal, and TIH were similar between the V group (using a flutter valve) and the group using a conventional water-seal system. The tested valve system did not exhibit any adverse events and was proven to be safe.
